# Isolated Gluteal (Buttock) Pain Due to L2-3 Degenerative Disc Disease: A Case Report Highlighting Diagnostic Challenges and the Role of Spinal Injection in Guiding Management

**DOI:** 10.7759/cureus.112950

**Published:** 2026-07-19

**Authors:** Hazem Alkatreeb, Laila Metwaly, El Fatih Bashir El Malik

**Affiliations:** 1 Neurosurgery, Al-Ahli Hospital, Doha, QAT; 2 Neurology, Independent Practice, New York, USA

**Keywords:** buttock pain, diagnostic injection, differential diagnosis, high lumbar radiculopathy, l2-3 disc degeneration, transforaminal lumbar interbody fusion

## Abstract

High lumbar degenerative disc disease (DDD) is an uncommon cause of radiculopathy and may present with atypical clinical features, creating diagnostic uncertainty and delaying appropriate management. We report the case of a 48-year-old man who presented with chronic isolated right gluteal pain without lower limb symptoms or sciatic radiation. The initial differential diagnosis included sacroiliac joint dysfunction and piriformis syndrome. Lumbar imaging demonstrated advanced L2-3 degenerative disc disease with severe foraminal stenosis. Because of the unusual presentation, a selective L2-3 transforaminal epidural steroid injection was performed as a diagnostic and therapeutic procedure, resulting in marked temporary pain relief and confirming the symptomatic level. Following recurrence of symptoms, the patient underwent L2-3 transforaminal lumbar interbody fusion with decompression and instrumentation, with significant postoperative improvement. This case highlights that isolated buttock pain may represent an atypical manifestation of upper lumbar radiculopathy and emphasizes the value of targeted diagnostic injections in identifying the pain generator before definitive surgical treatment.

## Introduction

Lumbar degenerative disc disease (DDD) is one of the most common causes of chronic low back pain and lumbar radiculopathy, most frequently affecting the lower lumbar levels. Compared with lower lumbar degenerative disease, upper lumbar pathology involving the L1-L3 levels, particularly the L2-3 level, is considerably less common and accounts for only a small proportion of lumbar degenerative pathology, contributing to its relative underrecognition in clinical practice [1,2]. In contrast, upper lumbar degenerative disease, particularly at the L2-3 level, is relatively uncommon and may present with atypical clinical manifestations, making diagnosis challenging and potentially delaying appropriate treatment [1,2].

Lumbar radiculopathy classically presents with dermatomal lower extremity pain accompanied by sensory disturbances or motor weakness. However, upper lumbar nerve root pathology may produce less typical symptoms, including isolated gluteal pain without associated lower limb radiation or neurological deficits, often leading to an initial diagnosis of sacroiliac joint dysfunction, facet-mediated pain, piriformis syndrome, or other musculoskeletal conditions [3,4]. Anatomically, the posterior rami of the upper lumbar nerve roots contribute to the superior cluneal nerves, providing a plausible explanation for referred gluteal pain in patients with upper lumbar pathology [1,5,6].

Selective transforaminal epidural steroid injections may serve not only as a therapeutic intervention but also as an important diagnostic tool for confirming the symptomatic level in patients with atypical clinical presentations. Accurate identification of the pain generator is essential before considering definitive surgical treatment in patients with degenerative lumbar disease.

This report describes a patient with isolated chronic gluteal pain caused by L2-3 foraminal stenosis who lacked the classical features of lumbar radiculopathy. The case highlights the diagnostic value of selective transforaminal injection in confirming the symptomatic level and emphasizes the importance of considering upper lumbar pathology in the differential diagnosis of isolated buttock pain before definitive surgical management.

## Case presentation

A 48-year-old man presented with a long-standing history of chronic low back pain localized exclusively to the right gluteal region. The pain was not associated with lower extremity radiation, sensory disturbances, motor weakness, or other neurological deficits. The patient reported chronic baseline pain ranging from 3-4/10, with recurrent exacerbations reaching 9-10/10 during physical activity, particularly with lumbar flexion. Physical examination demonstrated painful restriction of the lumbar range of motion, predominantly during forward flexion. Straight-leg raise testing was negative (80°), and no focal neurological deficits were identified. Sacroiliac provocative tests (Gaenslen, thigh thrust, distraction, compression, and sacral thrust) were predominantly negative. There was no focal point tenderness over the painful gluteal region, supporting a referred rather than a local pain generator. There was no anterior thigh pain, lower-extremity numbness, weakness, or radicular pain despite the advanced L2-3 pathology, emphasizing the atypical nature of this presentation. Before presenting to our service, the patient had already undergone extensive conservative management by another physician, including oral medications, physical therapy, local injections, and lower lumbar facet joint injections targeting presumed facet-mediated pain and sacroiliac joint dysfunction, none of which resulted in significant symptomatic improvement. Because of the atypical presentation, the patient underwent extensive conservative management at several healthcare facilities.

Given the persistent symptoms, lumbar spine imaging was obtained. Magnetic resonance imaging (MRI) demonstrated advanced degenerative disc disease at the L2-3 level with marked disc height reduction and severe foraminal stenosis (Figure 1). Computed tomography (CT) further confirmed disc space narrowing, osteophyte formation, and severe foraminal narrowing at the same level (Figure 2). Three-dimensional CT reconstruction clearly illustrated the extent of the degenerative changes and foraminal compromise (Figure 3).

**Figure 1:**
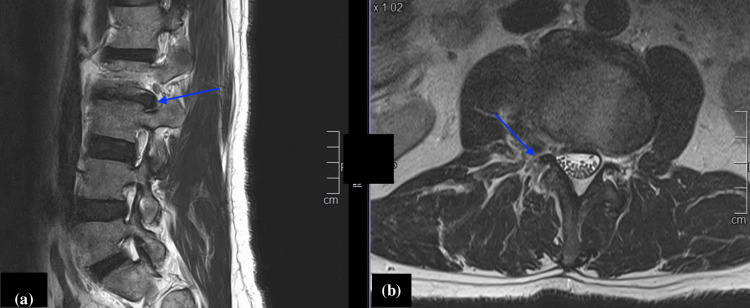
Preoperative MRI demonstrating advanced L2-3 degenerative disc disease with foraminal stenosis Preoperative T2-weighted sagittal (a) and axial (b) MRI images of the lumbar spine demonstrating advanced degenerative disc disease at the L2-3 level with disc height reduction, signal changes, and associated foraminal stenosis. Blue arrows indicate the L2-3 degenerative lesion and the corresponding foraminal stenosis responsible for the patient's symptoms.

**Figure 2:**
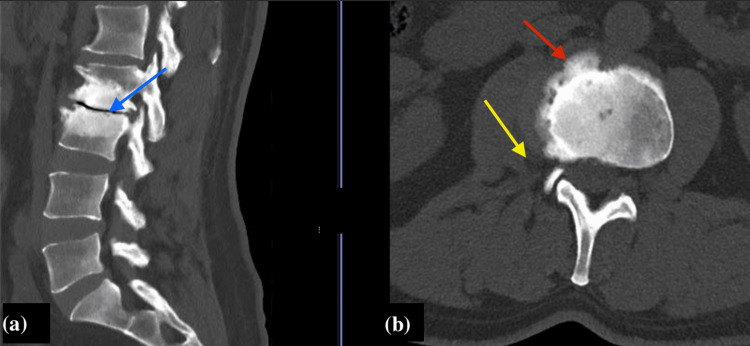
Preoperative CT demonstrating advanced L2-3 degenerative disc disease with foraminal stenosis Preoperative sagittal (a) and axial (b) CT images of the lumbar spine demonstrating advanced L2-3 degenerative disc disease. The blue arrow indicates the narrowed intervertebral disc space, the yellow arrow highlights the severe L2-3 foraminal stenosis, and the red arrow indicates the associated osteophyte formation.

**Figure 3:**
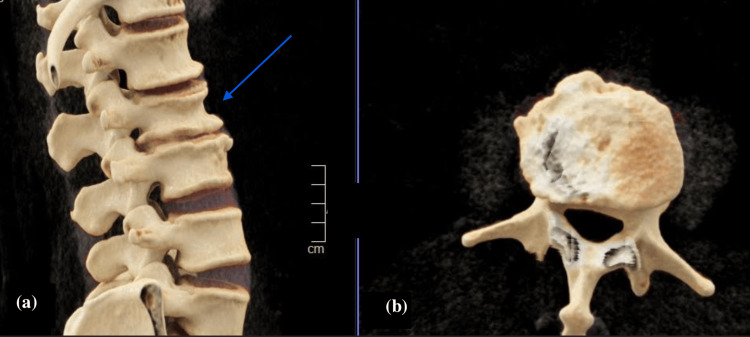
Three-dimensional CT reconstruction demonstrating advanced L2-3 degenerative disc disease Preoperative three-dimensional CT reconstruction sagittal (a) and axial (b) images illustrating advanced L2-3 degenerative changes. The blue arrow highlights the degenerative lesion at the L2-3 level.

Despite these radiological findings, the patient's isolated gluteal pain, without classic lower-extremity radicular symptoms, created diagnostic uncertainty. After unsuccessful treatment with oral medications, physical therapy, and previous spinal injections, a selective right L2-3 transforaminal epidural steroid injection was performed as both a diagnostic and therapeutic intervention. The injection resulted in significant symptomatic improvement for approximately two months before recurrence of symptoms, confirming the L2-3 level as the pain generator and supporting the decision to proceed with definitive surgical management.

Following the recurrence of symptoms several weeks later, definitive surgical management was recommended. The patient underwent L2-3 transforaminal lumbar interbody fusion (TLIF) with decompression and instrumentation. Postoperative radiographs demonstrated satisfactory instrumentation, appropriate interbody cage placement (Figure 4), restoration of disc height, and satisfactory spinal alignment (Figure 5). The postoperative course was uneventful, and the patient experienced marked clinical improvement. At approximately one year of follow-up, the patient remained symptomatically improved, had returned to his usual work, and continued to do well clinically.

**Figure 4:**
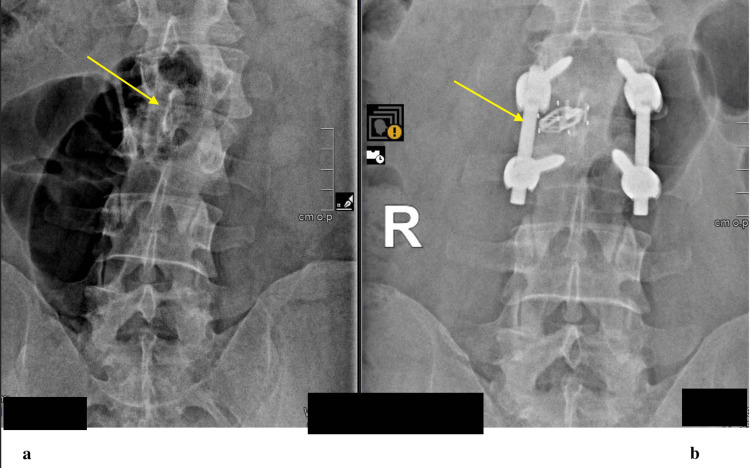
Preoperative and postoperative anteroposterior lumbar spine radiographs following L2-3 transforaminal lumbar interbody fusion Anteroposterior (AP) radiographs of the lumbar spine. (a) Preoperative image demonstrating degenerative changes at the L2-3 level. The yellow arrow identifies the affected level. (b) Postoperative image demonstrating pedicle screw fixation and interbody cage placement at L2-3. The yellow arrow highlights the pedicle screw construct used for stabilization of the L2-3 spinal segment.

**Figure 5:**
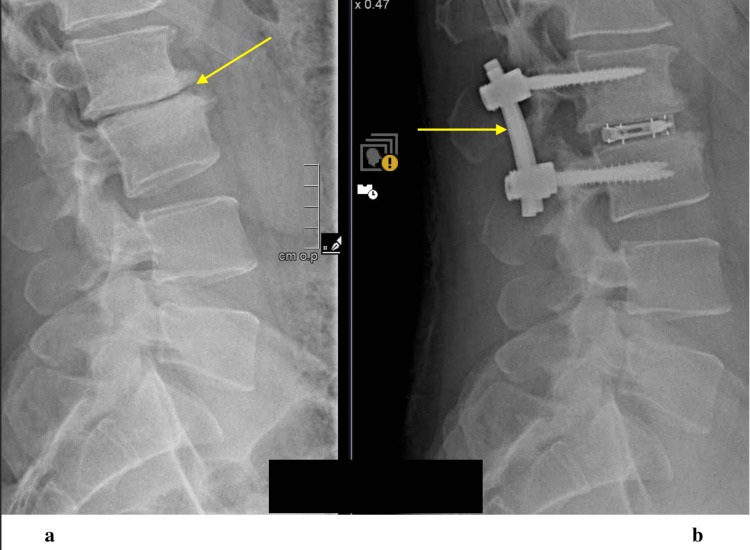
Preoperative and postoperative lateral lumbar spine radiographs following L2-3 transforaminal lumbar interbody fusion Lateral X-ray images of the lumbar spine. (A) Preoperative image demonstrating degenerative disc disease with reduced disc height at the L2-3 level. The yellow arrow identifies the affected level. (B) Postoperative image demonstrating restoration of disc height following L2-3 transforaminal lumbar interbody fusion (TLIF). The yellow arrow highlights the pedicle screw instrumentation used for stabilization of the L2-3 spinal segment.

## Discussion

Upper lumbar radiculopathy represents an uncommon clinical entity and may present with atypical manifestations that differ substantially from the classic pattern of lower lumbar radiculopathy. This case illustrates isolated buttock pain as the sole presenting symptom of L2-3 foraminal stenosis, emphasizing how upper lumbar pathology can easily be overlooked or misdiagnosed in the absence of typical lower extremity radicular symptoms. Similar atypical presentations involving the gluteal region have increasingly been recognized in the literature, highlighting the importance of maintaining a broad differential diagnosis when evaluating patients with persistent buttock pain [1,2].

The differential diagnosis of isolated buttock pain is extensive and includes sacroiliac joint dysfunction, facet-mediated pain, piriformis syndrome, myofascial pain syndromes, superior cluneal neuralgia, and superior gluteal neuralgia. However, accumulating evidence suggests that gluteal pain may represent an atypical manifestation of lumbar radiculopathy rather than an isolated musculoskeletal disorder [1,5]. In particular, the presence of gluteal trigger points has been shown to correlate significantly with lumbosacral nerve root involvement, supporting the concept that referred buttock pain may originate from underlying nerve root pathology rather than local soft tissue disease [5]. In the present case, the patient's pain was confined to the medial gluteal region near the sacroiliac joint; sacroiliac joint provocative tests were predominantly negative; there was no focal point tenderness over the painful region; and previous local and lower lumbar facet joint injections had failed to provide symptomatic relief. These findings, together with the radiological evidence of advanced L2-3 foraminal stenosis, supported pursuing a selective L2-3 transforaminal epidural steroid injection as the most appropriate diagnostic intervention while maintaining a broad differential diagnosis.

The anatomical basis for this presentation is likely related to the contribution of the posterior rami of the upper lumbar nerve roots (L1-L3) to the superior cluneal nerves. Irritation of these nerve branches may produce non-dermatomal referred pain confined to the gluteal region without the classical anterior thigh or lower limb symptoms expected in lumbar radiculopathy. Experimental and mechanistic studies further support this explanation by demonstrating the role of dorsal ramus irritation in generating gluteal pain [4,6].

Distinguishing radicular from non-radicular causes of buttock pain remains clinically important because treatment strategies differ substantially. Previous studies have demonstrated that selective nerve root blocks can serve as valuable diagnostic tools for differentiating lumbar radiculopathy from facet-mediated or referred pain, particularly in patients with spinal stenosis and atypical clinical presentations [6].

Although conservative treatment remains the first-line approach for most patients with lumbar degenerative disease, clinicians should remain vigilant for red-flag findings, including bowel or bladder dysfunction, saddle anesthesia, or progressive neurological deficits suggestive of cauda equina syndrome, which require urgent surgical evaluation.

In the present case, MRI and CT imaging demonstrated advanced L2-3 degenerative disc disease with severe foraminal stenosis. Nevertheless, imaging findings alone were insufficient to establish the symptomatic level because the patient's clinical presentation lacked the classical features of lumbar radiculopathy. A selective transforaminal epidural steroid injection provided both diagnostic confirmation and temporary symptomatic relief, thereby identifying the affected nerve root and supporting subsequent surgical decision-making [5].

Although definitive treatment ultimately required transforaminal lumbar interbody fusion, the principal educational value of this report lies in the diagnostic pathway rather than the surgical procedure itself. Similar reports have shown that upper lumbar disc pathology may produce atypical distal pain patterns, resulting in diagnostic confusion when upper lumbar radiculopathy is not considered early in the evaluation process [1]. Unlike most previously reported cases, which were associated with anterior thigh pain, lower-extremity symptoms, or neurological findings, our patient presented exclusively with isolated gluteal pain despite advanced L2-3 foraminal stenosis. This distinctive presentation further emphasizes the diagnostic challenge and educational value of recognizing atypical upper lumbar radiculopathy.

Although the patient's subsequent response supported upper lumbar radiculopathy as the symptomatic pain generator, alternative causes of localized gluteal pain, including superior cluneal neuralgia and superior gluteal neuralgia, should always be considered during the diagnostic evaluation. In this patient, the clinical presentation, previous unsuccessful local injections, predominantly negative sacroiliac provocative tests, absence of focal tenderness, imaging findings, and diagnostic response to the selective L2-3 transforaminal injection collectively favored L2-3 pathology while emphasizing the importance of systematic exclusion of other pain generators.

Overall, this case highlights the importance of considering upper lumbar radiculopathy in patients presenting with isolated buttock pain despite the absence of classical lower limb symptoms. A systematic approach combining careful clinical assessment, appropriate imaging, and targeted diagnostic injections remains essential for accurate diagnosis and timely definitive management. The purpose of this report is not to suggest that isolated gluteal pain should routinely be attributed to upper lumbar radiculopathy, but rather to emphasize that upper lumbar pathology should be included in the differential diagnosis of patients presenting with localized gluteal pain without classic lower-extremity symptoms.

## Conclusions

This case highlights the importance of considering upper lumbar radiculopathy as part of the differential diagnosis in patients presenting with isolated buttock pain despite the absence of classic lower-extremity radicular symptoms. Severe L2-3 foraminal stenosis may manifest with atypical clinical features, resulting in diagnostic uncertainty and potential misattribution to more common musculoskeletal conditions. Careful clinical assessment, appropriate imaging, consideration of alternative pain generators, and targeted diagnostic injections remain essential before attributing symptoms to upper lumbar radiculopathy and proceeding with definitive surgical intervention. Although the findings are based on a single case, they emphasize the educational value of recognizing this uncommon presentation and incorporating upper lumbar pathology into the differential diagnosis of localized gluteal pain to facilitate timely diagnosis and appropriate management.
